# Novel PSEN1 (P284S) Mutation Causes Alzheimer's Disease with Cerebellar Amyloid β-Protein Deposition

**DOI:** 10.2174/1567205019666220718151357

**Published:** 2022-11-10

**Authors:** Mingrong Xia, Chenhao Gao, Huayuan Wang, Junkui Shang, Ruijie Liu, Yang You, Weizhou Zang, Jiewen Zhang

**Affiliations:** 1 Department of Neurology, Zhengzhou University People’s Hospital, Henan Provincial People’s Hospital, Zhengzhou 450003, Henan, China;; 2 Department of Neurology, Henan University People’s Hospital, Henan Provincial People’s Hospital, Zhengzhou 450003, Henan, China;; 3 Academy of Medical Sciences, Zhengzhou University, Zhengzhou 450003, Henan, China;; 4 Department of Radiology, Zhengzhou University People’s Hospital, Henan Provincial People’s Hospital, Zhengzhou 450003, Henan, China

**Keywords:** Alzheimer's disease, PSEN1, P284S mutation, ^18^F-florbetapir (AV-45) PET, cerebellar Aβ deposition, cerebral cortex

## Abstract

***Background/Objective*:** AD-associated PSEN1 mutations exhibit high clinical heterogeneity. The discovery of these mutations and the analysis of their associations with cases such as EOAD should be critical to understanding AD's pathogenesis.

***Methods*:** We performed clinical analysis, neuroimaging, target region capture and high-throughput sequencing, and Sanger sequencing in a family of 3 generations. The underlying Alzheimer’s pathology was evaluated using biomarker evidence obtained from cerebrospinal fluid (CSF) amyloid testing and ^18^F-florbetapir (AV-45) PET imaging.

***Results*:** Target region capture sequencing revealed a novel heterozygous C to T missense point mutation at the base position 284 (c.850 C>T) located in exon 8 of the PSEN1 gene, resulting in a Proline-to-Serine substitution (P284S) at codon position 850. The mutation was also identified by Sanger sequencing in 2 family members, including proband and her daughter and was absent in the other 4 unaffected family members and 50 control subjects. Cerebrospinal fluid (CSF) amyloid test exhibited biomarker evidence of underlying Alzheimer’s pathology. ^18^F-florbetapir (AV-45) PET imaging indicated extensive cerebral cortex and cerebellar Aβ deposition.

***Conclusions*:** We discovered a novel PSEN1 pathogenic mutation, P284S, observed for the first time in a Chinese family with early-onset AD.

## 1 INTRODUCTION

Alzheimer’s disease (AD) refers to a clinical spectrum of neurodegenerative disorders characterized by memory impairment, behavioral disturbances, and multiple cognitive deficits [1]. Genetic change is one of the primary risk factors for AD. Amyloid β-protein precursor (APP), presenilin 1 (PSEN1) and presenilin 2 (PSEN2) genes are known genetic causes of AD, especially early-onset familial Alzheimer's disease (EOFAD). Mutations in these three genes could enhance Aβ production and deposition [2, 3]. Over 300 PSEN1 gene mutations have been reported in EOAD patients, sug- gesting that it is the most common genetic determinant of EOAD (*http://www.alzforum.org/mutations/psen-1, accessed in August 2021*). To our knowledge, PSEN1 mutations are associated with AD and have high clinical heterogeneity. Identifying these mutations in EOAD patients and analyzing their roles in EOAD development should be essential to understanding AD's genetic mechanisms [4]. In this study, we described a novel mutation (c.850 C>T) in exon 8 of the PSEN1 gene (p.P284S) as an important cause of early-onset Alzheimer’s disease with cerebellar β-amyloid protein (Aβ) deposition. Cerebral spinal fluid (CSF) amyloid test showed that the level of Aβ42 and the Aβ42/Aβ40 ratio were decreased.^18^F-florbetapir (AV-45) PET imaging indicated extensive cerebral cortex and cerebellar Aβ deposition. Therefore, investigating the clinical characteristics of AD patients and the effect of PSEN1 gene mutation was crucial for understanding the genetically linked pathogenesis of AD.

## 2 MATERIALS AND METHODS

### 2.1 Subjects

**Fig. (1):**
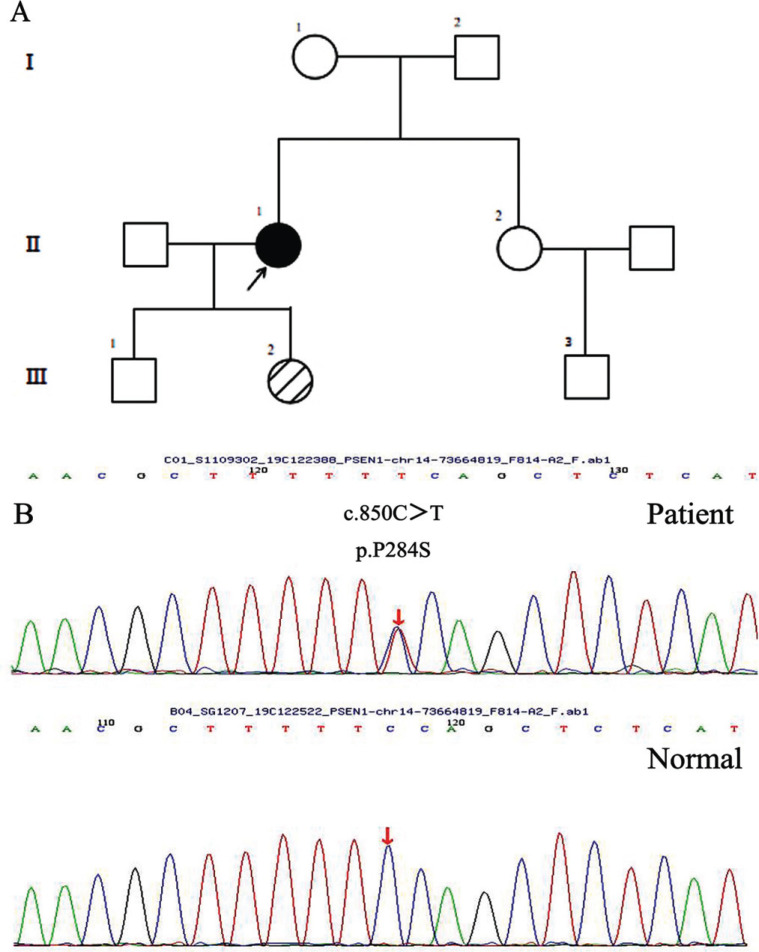
**(A) Pedigree of the early-onset familial Alzheimer’s disease with PSEN1 Pro284Ser mutation.** The arrow indicates the proband; circle, female; square, male; black symbol, affected family member; slashed symbol, mutation carrier. Blood samples were available from the proband (II: 1) and family members I: 1, I: 2, II: 2, III: 1, III: 2. **(B) DNA sequencing chromatograph of exon 8 of the PSEN1 gene.**

The proband (II:1) was clinically diagnosed with possible AD at the Department of Neurology, Zhengzhou University People’s Hospital, according to the criteria recommended by the National Institute on Aging-Alzheimer’s Association (NIA-AA) [5]. The family of the proband, consisting of three generations, came from the central and southern part of Henan province, China (Fig. **1**). The proband and other five family members were enrolled, and 50 control subjects were recruited to participate in this study.

Conventional brain magnetic resonance imaging (MRI) was carried out to confirm the diagnosis and rule out other differential diagnoses. Brain ^18^F-florbetapir (AV-45) PET imaging [6] and CSF amyloid test were performed in one patient (II:1). The final diagnosis was established following the current clinical diagnostic criteria for AD.

### 2.2 Target Regions Capture and Analysis

Blood samples were also collected from the proband (II: 1), the proband’s family members (I: 1, I: 2, II: 2, III: 1, and III: 2), and 50 unrelated healthy controls. Informed consent was obtained from all subjects. The study protocol was approved by the Institutional Review Board of Zhengzhou University People’s Hospital.

We designed 60mer biotin-labeled probes that bind to the exon of the target region. First, we prepared the library for the Illumina platform sequencing. The target gene regions were captured using the GenCap Kit (MyGenostics Inc., Beijing, China). Then, high throughput sequencing was performed using Illumina NextSeq 500 and bioinformatics analysis. Briefly, the library was prepared using the library preparation kit (MyGenostics Inc.) for the Illumina platform requirements. About 1-5 μg DNA sample was enzymatically fragmented. The enzymatic fragmentation of genomic DNA was end-repaired, and adapters were added. The length of the prepared libraries was about 350-400bp, and the template was amplified in a thermal cycler, and then the amplicon was analyzed using Agilent 2100 Bioanalyzer. After hybridization with the biotin-labeled probe under certain conditions, streptavidin-modified magnetic beads were used to tag and covalently bind the biotin-labeled probe to capture the target gene. Finally, magnetic adsorption beads carrying the target gene were washed, the DNA was eluted, and amplified. The libraries were ready for massively parallel sequencing. Illumina uses a unique bridge amplification reaction. The library was loaded onto the sequencing flowcell of the NextSeq 500 sequencer. Two types of fluorescent-labeled nucleotides were sequenced using a reversible sequencing-by-synthesis reaction. Each cycling reaction was only extended to a correct complementary base. The bases were confirmed based on four different fluorescence signals to ensure the quality of the final nucleic acid sequence. After several cycles, the nucleic acid sequence was completely read. The obtained data were filtered, separated, compared with the reference sequence, and annotated to obtain information on the mutation and its biological significance. Subsequently, the identified single nucleotide polymorphism was determined using the NCBI dbSNP137, HapMap, 1000 human genome dataset (released on 05/21/2011; http://www.1000genomes.org/), and ExAC Browser Beta (http://exac.broadinstitute.org/). The pathogenicity of the single nucleotide mutation was predicted using SIFT, Polyphen2, GREP+, and Mutation Taster.

### 2.3 Cerebrospinal Fluid Biomarker Analysis

Cerebrospinal fluid (CSF) was obtained by lumbar puncture in the morning. The CSF was centrifuged at 2000x g for 10 min, and the supernatant was collected and stored at -80 ^o^C until analysis. Amyloid-beta 1-42 (Aβ1-42), amyloid-beta 1-40 (Aβ1-40), total tau protein (t-tau), and phosphorylated-tau at T181 protein (p-tau) were quantified using a solid-phase enzyme-linked immune absorbent assay (Fujirebio, Gent, Belgium) according to the manufacturer’s instructions. Briefly, the antigen-specific monoclonal antibodies were coated onto the 96-well plate; human CSF was added and incubated with a biotinylated secondary antibody, followed by the detection of antigen-antibody complex by a horseradish peroxidase-labeled streptavidin. After the addition of the substrate, samples developed a color. The color intensity was measured to detect protein in the CSF sample.

### 2.4 Sanger Sequencing

To validate the DNA sequence variants detected by Sanger sequencing, we performed a polymerase chain reaction (PCR). The primers for the target site where the variant was located in the PSEN1 gene were designed. The PCR primers were the forward 5’-ATTCCTCCCTACCACC-CATT-3’ and the reverse 5’-CCAGGAATGCTGTGCA-TTTA-3’. The PCR amplification was performed on a Multigene OptiMix thermal cycler (Labnet, Edison, NJ, USA). After PCR amplification, PCR products of the proband and the other seven family members were purified and directly sequenced on an ABI3100 automated sequencer (Applied Biosystems, Foster City, CA, USA). Sequencing reads and lists were compared using Chromas software and NCBI blast.

## 3 RESULTS

### 3.1 Case Reports

**Fig. (2):**
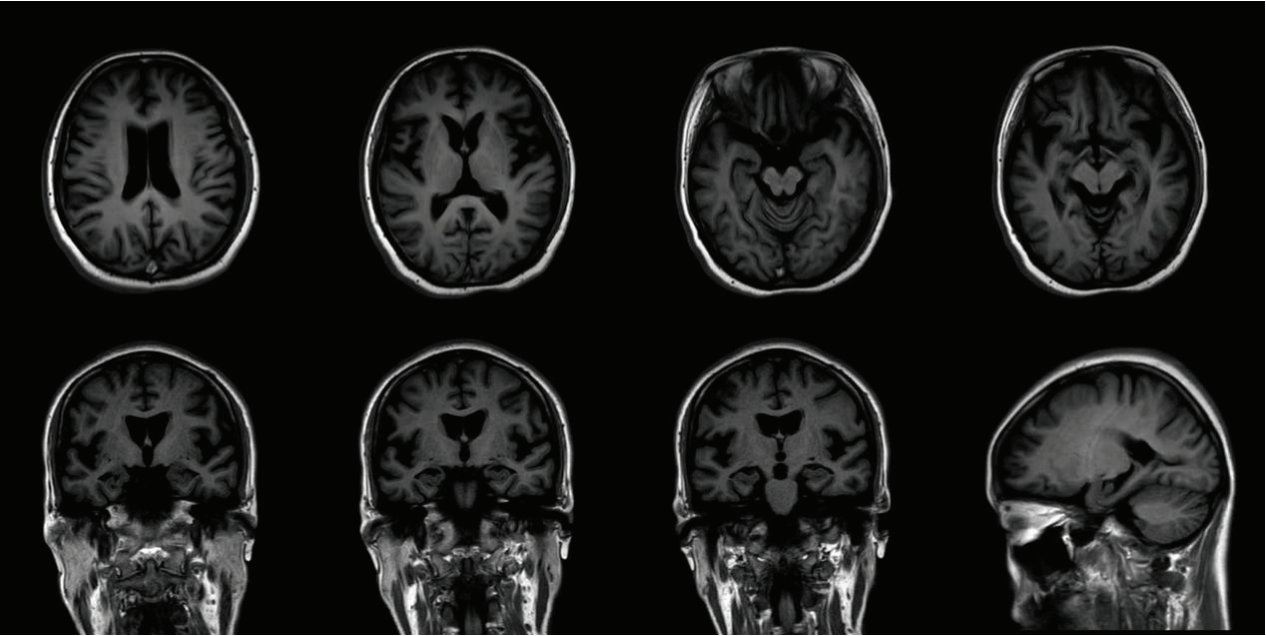
**The corresponding MRI scans.** MRI revealed diffuse cortical atrophy in the temporal lobe and hippocampal atrophy.

**Fig. (3):**
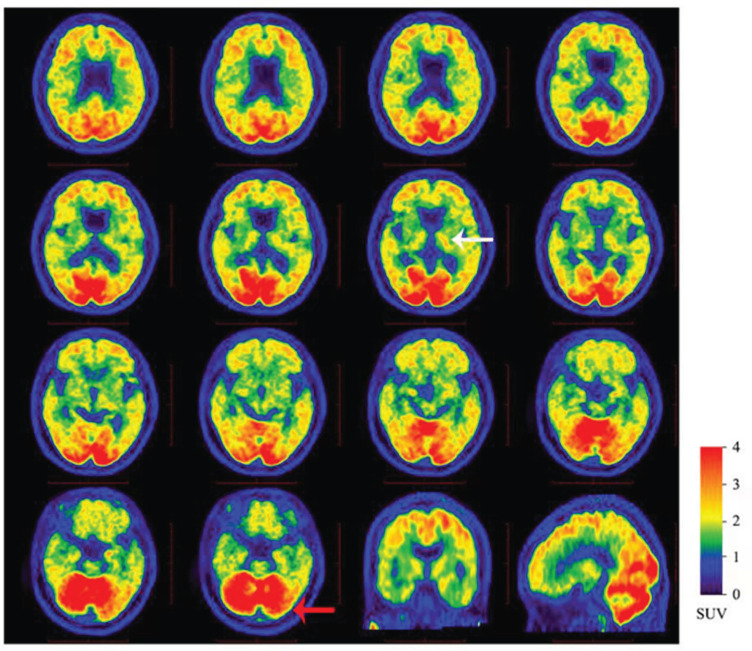
**The corresponding ^18^F-florbetapir (AV-45) PET scans.** ^18^F-florbetapir (AV-45) PET shows extensive cerebral cortex and cerebellar Aβ deposition. The white arrow indicates that there is a certain amount of Aβ deposition in the putamen. The red arrow indicates a large amount of Aβ deposition in the cerebellum. **Abbreviations:** MRI, Magnetic resonance image; PET, positron emission tomography; SUV, standardized uptake value.

The proband (II:1) was a 37-year-old female who presented with initial depression, drooping shoulders and a slightly forward and semi-sagging forearm. The symptoms started at the age of 30 years. One year later, she had memory loss, especially short-term memory, followed by glassy eyes, slow movement, and sluggish response. These clinical symptoms gradually worsened. Personality changes and abnormal behaviors characterized by increased mood swings, irritability, crying, drooling, and lack of interest were seen since age 34. Subsequently, the speech was also significantly impacted and progressed worse. She just communicated with her family members, not with others. As time progressed, she was reluctant to go out and had apparent memory impairment and significant cognitive decline. At the age of 36, these symptoms were significantly worse than before, and she experienced slower and unsteady walking, wider strides, right-leaning when walking, and leaning back when sitting, clumsy movements, and increased muscle tension in her limbs, but without seizures. She needed many attempts to complete a certain movement. Until now, she is still alive and almost lost the ability to care for herself and was completely reliant upon her husband and parents for the daily basic activities of living. She also presented slightly decreased facial expressions, combined with slowness, instability, and rigidity, which suggested Parkinson’s syndrome. She was treated accordingly. Neurological examination revealed memory impairment, cognitive deficits, cerebellum symptoms and ataxia, and pyramidal and extrapyramidal signs. She had a Mini-Mental State Examination (MMSE) score of 23/30 at the age of 34 years. Magnetic resonance images (MRI) were performed at her 34 years and revealed mild cortical atrophy in the temporal lobe and hippocampus atrophy (Fig. **2**). CSF amyloid test on the proband showed that the levels of Aβ42 and Aβ42/Aβ40 ratio were decreased, and pathological phospho-tau and total-tau were increased, respectively (Table **1**). ^18^F-florbetapir (AV-45) PET imaging showed extensive cerebral cortex and cerebellar Aβ deposition (Fig. **3**).

### 3.2 Mutation Analysis

Target region capture sequencing yielded a novel and heterozygous missense mutation (c.850C>T, p.Pro284Ser) (Fig. **1**) in the proband who had the clinical manifestation of Alzheimer’s disease. The mutation was located on exon 8 of the PSEN1 gene, which was associated with EOAD. Although this missense mutation was reported in an early onset AD [7], The pathogenicity analysis of the p.P284S mutation was not found according to the AD & Frontotemporal Dementia (FTD) mutation database (http://www.molgen.ua.ac.be/admutations/) or in The Human Genetics mutation database (HGMD, http://www.hgmd.org). Our report was the first to state the association of PSEN1 mutation with early-onset AD in China. Additionally, the pathogenicity of the mutation was predicted by using dbSNP and or the Exome Variant Server (http://evs.gs.washington.edu/EVS/). Furthermore, SIFT, Polyphen2, GREP+, and MutationTaster predicted the mutation damage and disease manifestation. These results demonstrated that the novel missense mutation was the most probable cause of the EOAD associated with cerebellum effect and Parkinson’s syndrome.

Through direct DNA sequencing analysis and complete coding sequence of the PSEN1 gene, we identified a heterozygous missense mutation in the first position of codon 284 in exon 8 (c.850C>T), resulting in Pro-to-Ser substitution (p.P284S) in the proband(II:1), and 1 member (III:2) of her family. But the mutation was not seen in the other 4 members of her family and 50 healthy controls.

## 4 DISCUSSION

We identified a novel missense mutation in exon 8 of the PSEN1 gene in a family clinically diagnosed with EOAD. To the best of our knowledge, this mutation was observed for the first time in a Chinese Han family with EOAD. The Pro284Ser mutation that was pathogenic in nature was supported by the residue of phylogenetic conservation. Although only two studies related to PS1 mutation at residue 284, including P284S [7] and P284L [8], were described previously, no studies reported increased deposits of cerebellar Aβ and the biomarker changes in cerebrospinal fluid in patients with PS1-P284S mutation. On the other hand, according to the guidelines recommended by the American College of Medical Genetics and Genomics(ACMG) [9], this novel PSEN1 p.P284S variant can be classified as pathogenic (supported by the evidence from PS2, PS4, PM1, PM2, PM5, and PP3). Furthermore, SIFT, Polyphen2, GREP+, and Mutation Taster predicted the disease manifestation and damage. These results predicted that the novel missense mutation was the pathogenic cause of dementia in this family.

We documented a family in which two members (II: 1 and III: 2) showed a genotype of Pro284Ser. However, only the index patient (II: 1) had the clinical phenotype characterized by memory impairment, cognitive deficits, cerebellum symptoms and ataxia, and Parkinson’s syndrome without seizures and had deposition of Aβ mainly in the cerebellum. Patients with the reported P284S and P284L mutations and other PSEN1 gene mutations at nearby sites were associated with the pyramidal system, spastic paraparesis, prominent MRI white matter hyperintense lesions [7], and cerebral microhemorrhage [8], except for memory loss and cognitive deficits. All this evidence makes this PS1 P284S mutation unique. The patients with the reported P284S and P284L mutations had the onset age ranging between 32-45 years, but in the proband, the onset was at 30 years with memory impairment. These findings and the previously described cases indicated that AD accompanied by PSEN1 P284S mutation showed increased heterogeneity. However, the phenoltypic heterogeneity caused by the residue 284 mutant of PSEN1 was still unclear. Most remarkably, the daughter of the proband, who was six years old, carried the P284S mutation. Long-term follow-up and continuous observation are required to consider the proband at age 30-40 as a possible preclinical patient.

Lumbar puncture for CSF biomarker assessment was performed in the proband who exhibited not only pathological Aβ42 but also showed pathological phospho-tau and total-tau (Table **1**). The decreased degree of these pathological biomarkers of the P284S mutation is compatible with an AD signature based on the Alzheimer’s Disease Association biomarker and ATN biological diagnostic marker classification [10]. Meanwhile, we performed an ^18^F-florbetapir (AV-45) PET scan to evaluate Aβ deposition and investigate the relationship between PS1-P284S mutation and cerebellar dysfunction. The deposition of Aβ was extensively seen in the cerebral cortex, cerebellum and putamen, which might indicate the motor symptoms of cerebellar dysfunction.

Previous studies have found that Aβ is associated with Parkinson’s motor symptoms, and the reduction of CSF Aβ level can lead to gait disturbances with frozen gait and postural instability [11, 12]. Brain Aβ deposition was associated with decreased dopamine transporter uptake in the anterior putamen and ventral striatum [13]. Brain Aβ deposition and α-synuclein are the pathological basis of Parkinson’s disease dementia (PDD) [14]. Several studies have shown that a higher cortical Aβ score/burden is a risk factor for PDD [11]. Apart from cortical Aβ deposition, the striatum is considered to be related to cognition and behavior [15]. However, there are conflicting views on whether the deposition of Aβ at the striatum can result in PDD [16-18]. Kalaitzakis *et al.* found that striatum Aβ pathology was common in PDD, which might reflect the development of dementia [16]. In our study, extensive Aβ deposition in the cerebral cortex and putamen and the decreased level of CSF Aβ42 in the proband might be related to the pathogenesis of Parkinson’s syndrome.

While previous studies proposed that Aβ-related pathology might follow a temporal evolution, considered typical for AD. The different phases were characterized by the gradual participation of different brain regions. The research found that the neocortex was always the first brain region to develop Aβ deposits. Additionally, the deposition of Aβ was exclusively seen in the allocortex in phase 2. The deposits were also observed in the diencephalon and striatum in phase 3, in the brainstem in phase 4, and the cerebellum in phase 5 [19, 20]. While studies focused on patients with the PS1 mutation revealed that the Aβ deposition might not follow the above progression [21, 22]. A previous study found that numerous Aβ42-reactive plaques in the cerebellum of PS1-E280A patients had robust cerebellar pathology [23], which might be relevant to high cerebellar PS1 mRNA expression. Meanwhile, the index patient (II: 1) who manifested cerebellum effect and Parkinson’s syndrome and had the deposition of Aβ mainly in the cerebellum might be associated with selective vulnerability [24]. One possible explanation for the phenotypes of PSEN1 P284S mutation in our study that was different from other PS1 mutations (including P284S [7] and P284L [8]) was that a region such as a cerebellum was relatively unaffected across sporadic AD patients, and might only be vulnerable in EOAD cases with certain but not with other PS1 mutations. This outcome raises the possibility of different pathogenic pathways in EOAD with different PS1 mutations. The phenomenon in our study also considerably suggests that the determining features of selective vulnerability depend not only on the characteristics of particular regions but also on the disease's etiology. This suggestion would contribute to the varied clinical features in different subjects with these mutations and could, to a certain extent, explain differences between individuals with sporadic AD, those with PS1 mutations, and those with mutations in other proteins such as APP. Further studies about the functions of the novel mutation of P284S to illuminate its pathogenic mechanism of the Aβ deposition in the cerebellum are required.

There are some limitations to this study. First of all, no other family members were affected, and co-segregation of the P284S mutation in the dementia family could not be confirmed. Secondly, further evidence for AD pathology and clinical phenotypic heterogeneity would be provided if we could demonstrate the biomarker changes through autopsy verification.

## CONCLUSION

In conclusion, we described a novel mutation in exon 8 at codon 284 (P284S) of the PSEN1 gene in a Chinese family with EOAD associated with cerebellum effect and Parkinson’s syndrome with cerebellar β-Amyloid protein deposition. CSF biomarker changes demonstrated that the Aβ42 and Aβ42/Aβ40 ratio levels were decreased. ^18^F-florbetapir (AV-45) PET imaging showed extensive cerebral cortex and cerebellar Aβ deposition. The clinical characteristics and the effect of the mutation are therefore crucial for understanding the pathogenesis of AD.

## Figures and Tables

**Table 1:** Cerebrospinal fluid biomarker analysis.

**Items**	**Test Method**	**Results**	**Unit**	**Tips**	**Reference Interval**
Human CSF Aβ_1-42_	ELISA	245.69	pg/ml	↓	610.00-974.00(age 21-51)
Human CSF Aβ_1-40_	ELISA	13823.00	pg/ml	-	-
Human CSF t-tau	ELISA	761.85	pg/ml	↑	47.00-225.00(age 21-51)
Human CSF p-tau	ELISA	48.13	pg/ml	↑	19.66-45.67(age 18-44)
Aβ_1-42_/ Aβ_1-40_	-	0.02	-	↓	>0.10

**Note:** *AD signature:* Aβ_1-42_ <610.00pg/ml; t-tau >225.00pg/ml; p-tau>45.67pg/ml; Aβ_1-42_/ Aβ_1-40_ <0.1.

**Abbreviations:** Aβ: amyloid-β, CSF: cerebrospinal fluid, p-tau: phosphorylated tau, t-tau: total tau.

## Data Availability

Not applicable.

## LIST OF ABBREVIATIONS

AD: Alzheimer’s Disease
APP: Amyloid β-Protein Precursor
PSEN1: Presenilin 1
PSEN2: Presenilin 2
EOFAD: Early-Onset Familial Alzheimer's Disease
Aβ: β-Amyloid Protein
CSF: Cerebral Spinal Fluid
P-tau: Phosphorylated Tau
T-tau: Total Tau
DNA: Deoxyribonucleic Acid
PCR: Polymerase Chain Reaction
MMSE: Mini-Mental State Examination
MRI: Magnetic Resonance Imaging
PET: Positron Emission Tomography
SUV: Standardized Uptake Value
HGMD: Human Genetics Mutation Database
ACMG: American College of Medical Genetics and Genomics
